# Look-ahead fixations during visuomotor behavior: Evidence from assembling a camping tent

**DOI:** 10.1167/jov.21.3.13

**Published:** 2021-03-10

**Authors:** Brian Sullivan, Casimir J. H. Ludwig, Dima Damen, Walterio Mayol-Cuevas, Iain D. Gilchrist

**Affiliations:** 1 School of Psychological Sciences, University of Bristol, Bristol, UK; 2 Department of Computer Science, University of Bristol, Bristol, UK

**Keywords:** look ahead fixations, visuomotor coordination, natural tasks, eye movements, visual attention

## Abstract

Eye movements can support ongoing manipulative actions, but a class of so-called look ahead fixations (LAFs) are related to future tasks. We examined LAFs in a complex natural task—assembling a camping tent. Tent assembly is a relatively uncommon task and requires the completion of multiple subtasks in sequence over a 5- to 20-minute duration. Participants wore a head-mounted camera and eye tracker. Subtasks and LAFs were annotated. We document four novel aspects of LAFs. First, LAFs were not random and their frequency was biased to certain objects and subtasks. Second, latencies are larger than previously noted, with 35% of LAFs occurring within 10 seconds before motor manipulation and 75% within 100 seconds. Third, LAF behavior extends far into future subtasks, because only 47% of LAFs are made to objects relevant to the current subtask. Seventy-five percent of LAFs are to objects used within five upcoming steps. Last, LAFs are often directed repeatedly to the target before manipulation, suggesting memory volatility. LAFs with short fixation–action latencies have been hypothesized to benefit future visual search and/or motor manipulation. However, the diversity of LAFs suggest they may also reflect scene exploration and task relevance, as well as longer term problem solving and task planning.

## Introduction

There are a wide array of activities in daily life and studies of naturalistic visuomotor behavior have reflected this variety, including tasks like making tea or a sandwich, driving, games, and sports (Chase & Simon, 1973; Land & Lee, 1994; Land, Mennie, Rusted, 1999; Land & McLeod, 2000; Land & Hayhoe, 2001; Kato & Fukuda, 2002; Kim & Lee, 2006; Foerster et al., 2011; Tatler, Hayhoe, Land, & Ballard, 2011; Timmis, Turner, & Van Paridon, 2014). These natural activities are distinct from most laboratory-based vision studies, which tend to use briefly presented static two-dimensional stimuli, presented in a discrete and repetitive trial structure, and viewed with the participant's head fixed. First, natural activities tend to be longer, taking several minutes to complete behavior. Second, they typically require several actions in a sequence. Third, the objects and stimuli required for natural tasks are distributed about the world and require large head movements or whole-body movements to acquire them visually and for manual manipulation. Look ahead fixations (LAFs), that is, preview fixations of an upcoming task-relevant object before direct manipulation, have been shown to occur in these scenarios. In the current study, we detail the look ahead behavior while participants assembled a camping tent. This dataset was collected as a part of a larger project on real-world problem solving. LAFs have not been widely studied but seem to have a role in planning and are a visual behavior unaddressed in current models of visuomotor control. In this article, we describe LAF behavior in a task that consists of several subtasks extended over longer timescales than previous studies. We document several novel features of LAF behavior.

### Look ahead fixations

As defined by Pelz and Canosa (2001), LAFs are “fixations on objects not relevant to the immediate subtask, but relevant for a future subtask.” Importantly, this preview should be separated by at least one fixation elsewhere before an action starts, so that it is not simply a guiding fixation that lands on a target moments before the hand arrives. For example, during a complex task such as assembling a tent, a participant might look at the corner of the tent while putting together a support, then look elsewhere and then look back to the corner as they approach to insert the support. The information present in such a fixation could be used for planning, decreasing visuospatial uncertainty, getting object information to help with interaction, or it could be coincidental.

LAFs have been documented in natural tasks such as tea and sandwich making (Land et al., 1999; Hayhoe et al., 2003). Similar anticipatory behavior, where the eye regularly looks ahead of actions to gather information for the upcoming actions, has also been shown in musical sight reading, driving, and walking where the eye leads behavior (Land & Furneaux, 1997; Furneaux & Land, 1999; Lehtonen et al., 2013; Marigold & Patla, 2007; Wilkie, Wann, & Allison, 2008; Lehtonen et al., 2013).

Most relevant to our task, Pelz and Canosa (2001) described aspects of looking ahead when entering a bathroom and washing hands or filling a cup. They demonstrated that LAFs occur seconds before manipulation and can be considered distinct from guiding fixations. Further, they showed that task demands influenced LAF frequency, because participants who washed hands exhibited near three times as many LAFs. Mennie et al. (2007) extended these findings by examining LAF behavior in the context of a model building task that generated hundreds of LAFs. They replicated the finding that task demands influence LAF frequency and documented the distribution of LAF intervals and the rate of LAF versus look back fixations. They found that LAFs were about 20% of fixations made, were about 10 times more frequent than looking back, and the LAF latency distribution peaked at 2.5 seconds before the action occurred. Owing to experimental limitations, LAF intervals could only be considered up to 10 seconds because participants reused component areas in that task. Anecdotal evidence from Land et al. (1999) noted that a participant made a long latency look ahead (68 seconds), and when the eye returned later to fixate the object, saccade accuracy was poor. Further, they noted a short latency LAF (5 seconds) had a return saccade with high accuracy, suggesting that the quality of information gathered from a LAF decays over time or is subject to interference. Aside from this observation, there is no documentation of long latency LAFs, although preview fixations with long latencies have been studied in other domains as described elsewhere in this article.

The nature of the information conveyed by LAFs is not well-understood. Mennie et al. (2007) demonstrated in their task that LAFs influence future saccade accuracy and latencies, but not accuracy of hand movements. Aivar et al. (2005) showed in a virtual model copying task that altering the visual display covertly to swap model piece locations sometimes led to fixations on old preswap locations, suggesting that saccades were guided by spatial memory. Related experiments where participants navigated three-dimensional virtual environments have also shown memory for location and object identity. Although these experiments did not consider behavior in the context of LAFs, they did examine the effect of fixation preview on objects during visual search and change detection. Their results demonstrate the capacity for visuospatial memory to be built up over time in some contexts and suggest that LAFs might similarly influence behavior (Võ & Wolfe, 2012, 2015; Li et al., 2016, 2018; Kit et al., 2014). In complex sequential behavior, LAFs may indicate the degree of planning participants engage in, for example, looking at task-relevant items one, two, or more steps ahead. Interestingly, Forde et al.'s (2010) case study of an action disorganization syndrome patient found a complete deficit in LAFs, suggesting a role in planning. However, ultimately, we currently do not have a full picture of LAF behavior in extended tasks and how LAFs may relate to planning and coordination.

### Models of sequential action control

Although LAFs are of interest as a fundamental aspect of natural eye movement behavior, it is also worth briefly reviewing their relationship with models of sequential behavior. Human gaze is tightly linked to task demands and this has made generic computational models of real-world visual attention and behavior difficult to implement realistically, requiring a visuomotor agent with domain knowledge and means to achieve goal-oriented behavior. As a result, models of sequential behavior have tended to abstract away complexities and focus on generating plausible lists of action sequences, avoiding the simulation of a perceptual agent that acquires visual information to guide decision making and motor behavior.

The contention scheduling model (Cooper, Shallice, & Farringdon, 1995; Cooper & Shallice, 2000, 2006) is a hybrid symbolic–connectionist approach used to explain automated behavior as part of a hierarchal action sequencing system. Although the authors do not address fixation behavior, the model captures several elements of complex behavior relevant to our task. The model incorporates hierarchical action schemas (sets of action and subgoals sequenced to achieve an overall goal) that are in competition with one another and vie for activation. For example, in the task of making a cup of tea, an “add sugar” schema competes (at least in the UK) with the “add milk” schema, after the “add water: schema has run to completion. The evolution of these states of activation over time can be read out as an action sequence. Their work was based on interactive activation and competition connectionist networks (Grossberg, 1978; McClelland & Rumelhart, 1981; Rumelhart & McClelland, 1982) with elements of symbolic networks added to constrain how schemas and objects interact in physically plausible ways. The model's symbolic production system uses action primitives, state knowledge of the world (e.g., location of an object, is it open or closed, full or empty) and logical contingencies between schemas, objects and resources. Once parameters are tuned, the model can generate realistic motor sequences.

Botvinick and Plaut (2004; 2006) demonstrated an alternative approach using recurrent neural networks to produce plausible visuomotor sequences. This strategy has the advantage of using a network that learns from sequence data instead of hand tuning, and does not use assumptions of task constraints, schemas, or hierarchical representation. Their model predicts the steps of the tea and coffee making behavior as activations of a concatenated binary vector representing the current fixated object, current object held, and next action. The authors assume actions are directed toward objects that are selected by gaze and there is an obligatory coupling between fixation and manipulative actions. In this way, the model excludes LAFs, and are presumed absent in the training data. The authors later add noise to the network to simulate dysfunction and in principle this process could generate fixations to objects related to future task steps, but they would disrupt performance and lead to actions improperly timed in the task's progression.

Both models generate realistic sequences of labels of routine visuomotor behavior and can mimic elements of action disorganization syndrome. Both models are instructive but have limitations; contention scheduling requires hand coding of symbolic knowledge, whereas the recurrent network seems to primarily capture sequence statistics. Both models are abstracted to focus on action and goal sequences, but do not address the underlying visuomotor control problem.

An alternative approach considers sequential visuomotor behavior as a control problem that is solved via learning. For example, using a reinforcement learning approach (Sutton & Barto, 2018), Sprague and Ballard (2003) proposed a framework using visual routines, task-oriented image processing (Ullman, 1987; Hayhoe, 2000), where an agent learned three concurrent behaviors to follow a sidewalk, and approach and avoid objects in a virtual three-dimensional environment.

The agent has a set of task modules represented by independent *Q*-value functions that share the same action space. To select an action, the agent sums across *Q-*functions and chooses the action with the highest expected value. However, as in real life, there is uncertainty, and each task module has an independent estimate of the agent's location in the state space (represented as a Gaussian via a Kalman filter). In this context, foveating is operationalized as providing information to reduce uncertainty in this state space estimate. When a “fixated” module is selected to receive new information, all other modules propagate their Kalman filter state-space estimates forward without new data. The module to receive new visual information is chosen by estimating the difference in expected reward if a fixation were made versus not. The module that has the most expected loss is chosen to receive new visual information.

In this model, LAFs may arise when a module is selected to be fixated but the agent's navigation actions are determined by another subtask. Because the *Q*-functions are summed, typically the action chosen is a composite of task priorities, but in principle one module could dominate. Johnson et al. (2014) expanded on this approach without learning, instead using diffusion decision models to model task relevance and uncertainty in a driving simulation. Both architectures allow for LAFs to occur in the context of ongoing behavior, but the structure of the task (which involves a constant, limited set of continuous subtasks that are completed repeatedly) ensures that the lag between fixation and subsequent action will be relatively short. This behavior more closely resembles LAFs to feed the visual buffer (Land & Lee, 1994; Land & Furneaux, 1997; Furneaux & Land, 1999) than the long latency LAFs we describe elsewhere in this article.

Although the approaches as discussed could generate LAFs, none as originally proposed are well-suited to generating the variety we document in the current study. Recent advancements in deep learning and hierarchical reinforcement learning have demonstrated possible computational architectures that allow agents to learn complex behavior (Barto & Mahadevan, 2003; Botvinick, 2012; Botvinick & Weinstein, 2014). Advances in engineered systems using deep reinforcement learning have shown dramatic results for the control of simulated robotics and better than human performance in video games (Merel et al., 2018; Hessel et al., 2019; Vinyals et al., 2019). Although such models were not originally intended to model human behavior, they show that computational agents can solve complex tasks with human-level performance. These models provide an inspiration for psychological models and a candidate architecture for solving complex visuomotor coordination problems. We believe this avenue of research will be fruitful, but comparison to human behavior will require biological constraints and real-world generalization (Dulac-Arnold, Mankowitz, & Hester, 2019). Models that can simulate complex visuomotor behavioral sequences will allow investigations to document if they exhibit LAF behavior and under what conditions. If they do not, new theories will be needed with possible constraints or architecture changes to generate them.

### Current study

This work is part of a wider project examining real-world problem solving and developing assistive systems (UK Engineering and Physical Science Research Council GLANCE Project). During analysis of this dataset, novel LAF behavior was observed, prompting its use as a convenience sample. Although an unusual choice for an experimental task, assembling a tent provides a unique opportunity to study LAF behavior. It is a complex, nonroutine, real-world task where visuomotor behavior can be recorded while participants solve a complex problem in three-dimensional space. It taps into many aspects of cognition and perception, requiring the sequential assembly of several parts, and incorporates visual search, visuomotor guidance, and gross and fine motor skills.

LAFs may occur for several reasons during tent assembly. Some subtasks may not require foveal guidance, and task transitions may provide opportunities for LAFs to gather upcoming information. Setting up a tent is unique compared with prior experiments because the layout of the tent is unfamiliar and deformable. In hand washing or tea making, participants likely have considerable prior knowledge on scene layouts, and most items are static and in preset locations. While building a tent, the participant actively unpackages many items from a single bag and they regularly move around the environment. This means that there is likely a greater amount of spatial uncertainty in our task, which might be decreased by looking ahead. If LAFs reflect problem solving, one might expect a complex and uncommon task to encourage LAF behavior. If LAFs reflect action planning, the large number of steps involved in assembly and extended duration of the task should encourage their occurrence as participants may store upcoming task-relevant information in a “behavioral pipeline.” Some LAFs may be incidental owing to the physical salience of an object's image features, resulting in the eyes being drawn to that object regardless of its current or future task relevance. Incidental LAFs may also occur because the object has a future relevance (Fecteau & Munoz, 2006) that biases attention, but are not associated with planning or problem solving; this process is similar to elevated schema activation within Cooper and Shallice's (2000) framework where an action might occur prematurely.

We document several novel findings suggestive of LAFs being purposeful, targeting a variety of objects and occurring in a variety of subtasks. Additionally, we detail how LAF latencies (the time between a look ahead and related action) may be related to these purposes. Given the observational nature of our study, we cannot definitively distinguish LAFs supporting problem solving and motor planning from incidental LAFs owing to salience or task relevance, but we present numerous examples of LAFs in tent assembly that are likely to be purposeful.

In addition to LAF behavior, task complexity and the range of participant expertise provide an opportunity to describe visuomotor strategies and the variety of action sequences that occur. Capturing eye gaze and egocentric video data allow these elements of sequential visuomotor behavior to be catalogued and inform the future development of models of complex, sequential visual–motor behavior.

## Methods

### Participants

Twenty-three participants (12 female; mean age 23, years; range, 18–32 years) were recruited from Bristol, UK, and gave informed consent, including anonymized open data sharing. An additional participant initially gave consent and performed the study, but retracted later owing to privacy concerns.

### Materials

Participants completed a survey asking five questions on their experience with tents and camping: (1) How often do you camp each year? (2) Are you an experienced camper? (low/medium/high), (3) Do you own a tent? (yes/no) (4) How many times a year do you setup a tent? (5) How many times have you ever setup a tent?

**Figure 1.**
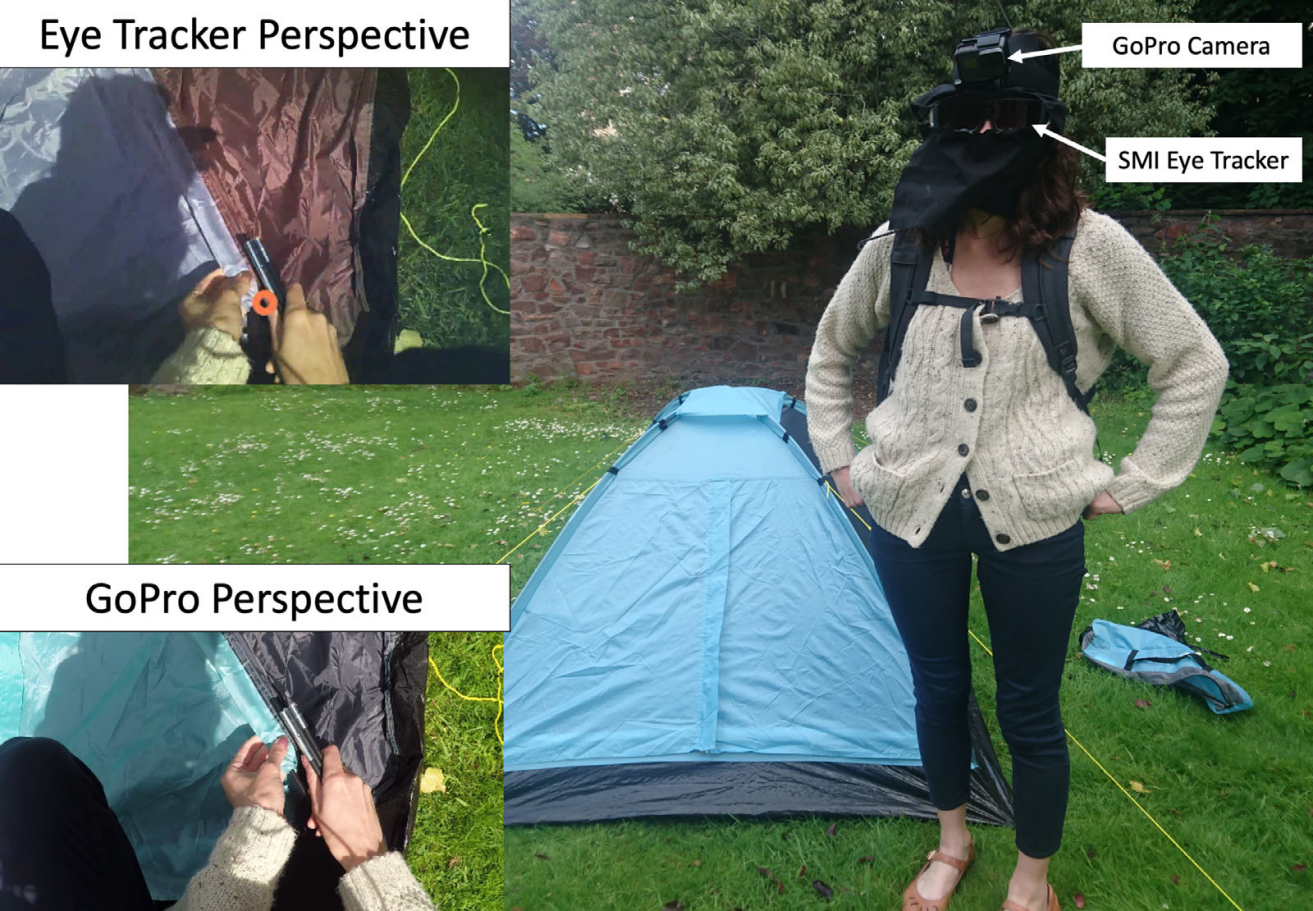
Participant standing next to a completed tent. Insets on left show example images from SMI Eye Tracker and GoPro. The participant wears a tracker with sunglass inserts and black fabric to block out infrared interference from the sun.

To track eye movements, participants wore a pair of SMI Eye Tracking Glasses, v1.8, 30hz binocular, scene camera resolution 1280 × 960 (field of view 60° × 46°), (SMI Gmbh, Berlin, Germany), as shown in Figure 1. We used the manufacturer's sunglasses insert to decrease infrared interference with eye images. Additionally, the tracker was fitted with black cloth on the top and bottom of the frame to further block out infrared interference. To capture high-resolution first-person video, participants also wore a head-mounted GoPro Hero 5, resolution 1920 × 1080 (field of view 123° × 94°). The eye tracker was calibrated initially indoors and then once again when the participant went outside. The experimenter viewed the track on a mobile recorder (Samsung Galaxy S3 with SMI ETG software) and judged by eye if tracking quality was sufficient. Quantitative measures of eye tracker accuracy were calculated post hoc.

Once calibrated, the participant proceeded to setup a two-person tent (Wilko 2-person dome tent; Wilko Retail Ltd. Workshop, UK) outdoors in a grassy area on the University of Bristol Campus. The participants could take as much time as they required and use the instructions as much or as little as desired.

The dataset can be found at https://sites.google.com/view/epic-tent (Jang et al., 2019); see the Appendix Shared Dataset for more information on the videos, datafiles, and annotations shared. Note, the dataset from Jang et al. (2019) differs from this study because it was amended with the withdrawal of one participant (noted elsewhere in this article) and the addition of six new participants’ data. The additional data were not part of our analysis.

### Analysis: Questionnaire

Real numbers were assigned to non-numeric responses on the questionnaire as follows: For the question “Are you experienced outdoors?”, a “0” was used to signify low experience, “0.5” as medium, and “1” as high. The question “Do you own a tent?” was turned into a binary value of “1” if “yes” and “0” if “no.” These were combined with numeric responses into a unified score as detailed in the Results.

### Analysis: Subtask labelling

The behavior can be described as a hierarchy of goals, tasks, and subtasks. Although all participants had the goal of successfully setting up the tent, the path of tasks and subtasks to achieve this is somewhat flexible; not all steps rely on one another. For simplicity, we refer to all annotated actions as subtasks. Subtasks were operationally defined as the set of common events in tent assembly whose beginning and end could be clearly annotated by an observer. A beginning was marked when a finger contacted an object to pick up, and endings marked when fingers released an object to put down. Other events were marked by the beginning and ending of manipulating an object for one task before being used for another. Note, instruction reading in participants without acceptable eye tracking was coded by periods when the participants held the instructions in front of them. See Table 1 for a list of subtasks and a diagram of the typical workflow in building a tent.

**Table 1.** Outline of steps in setting up a camping tent. (Left) Mid-level description of steps, local goals but no explicit description of perceptual-motor sequences required. (Center) High-level labels that concatenate several mid-level labels. These labels were used by human annotators (Right) Instruction headings given by the tent manufacturer; each included a short paragraph of text (not shown).

	Steps	Subtask label	Written instructions
1	Read instructions	(1) Read instructions	Self-explanatory
2	Pick up main bag	(2) Pick up/open/empty tent bag	(1) Layout the tent
3	Open main bag		
4	Empty main bag out		
5	Pick up tent	(3) Pick up/spread tent	
6	Spread tent out		
7	Pick up support pole bag	(4) Pick up/open/empty support pole bag	(2) Install the poles
8	Open support pole bag		
9	Empty support pole bag		
10	Pick up support pole #1	(5) Assemble support pole	
11	Assemble support pole #1		
12	Pick up support pole #2	(5) Assemble support pole	
13	Assemble support pole #2		
14	Insert support pole #1	(6) Insert Support Poles	
15	Insert support pole #2	(6) Insert Support Poles	
16	Insert support pole #1 into plastic tab 1	(7) Insert support pole into tabs	
17	Insert support pole #1 into plastic tab 2	(7) Insert support pole into tabs	
18	Insert support pole #2 into plastic tab 1	(7) Insert support pole into tabs	
19	Insert support pole #2 into plastic tab 2	(7) Insert support pole into tabs	
20	Pick up stake bag	(8) Pick up/open/empty stake bag	(3) Pegging the tent
21	Open Stake Bag		
22	Take Stakes		
23	Stake Corner 1	(9) Stake Corner	
24	Stake Corner 2	(9) Stake Corner	
25	Stake Corner 3	(9) Stake Corner	
26	Stake Corner 4	(9) Stake Corner	
27	Pick Up Guyline 1	(10) Place Guyline	(4) Place Guylines
28	Stake Guyline 1		
29	Pick Up Guyline 2	(10) Place Guyline	
30	Stake Guyline 2		
31	Pick Up Guyline 3	(10) Place Guyline	
32	Stake Guyline 3		
33	Pick Up Guyline 4	(10) Place Guyline	
34	Stake Guyline 4		
35	Tie Support Poles Above Vent	(11) Tie Support Poles Above Vent	Not Mentioned
36	Pick Up Vent Cover	(12) Pick Up/Attach Vent Cover	(6) Attach Vent Cover
37	Attach Vent Cover		

Subtask definitions are ambiguous; some subtasks could be combined into a compound task, or alternatively further subdivided. For example, in “assemble support,” the tent comes with two supports that are each disassembled into seven detached segments (approximately 50 cm long) with an elastic cord threaded between each segment. The participant must pick up the disassembled support and slide each of the segments together to form a whole (approximately 350 cm long). We consider picking up the support and assembling each segment to define the “assemble support” subtask. It would be possible to divide picking up and assembly, or even further divide the assembly of each segment of the support. Ultimately, for ease of annotation, we chose to define subtasks as object interactions that had a reasonably clear beginning and end of manipulation. Thus, “assemble support” was defined as from the moment of picking up the support, attaching all segments together, and then either putting the completed support on the ground or placing the support into the tent fabric.

We identified 37 steps required to build a tent (Table 1). One observer watched all videos marking the beginning and ending of each event, using the CinePlay annotation tool (Digital Rebellion, Los Angeles, CA). These annotations were turned into a frame-by-frame list of subtask labels.

Once all videos were labelled, the list was simplified down to 12 subtasks. This was possible because some subtasks use steps that could be grouped together; for example, the three steps of picking up, opening, and emptying the main bag out (main bag being the bag of bags containing all of the parts required), can be labelled as one act. Additionally, some subtasks are equivalent; for instance, the staking any of the tent corners of the tent was initially coded as a unique event, but it can also be considered as a repetition of a generic staking the corner subtask. Because there are several steps that required repetition, these were compressed into 12 generic labels (Table 1, center column). along with an “approach” label used when the participant was transitioning between subtasks, for example, walking around the tent to pick up an item.

### Analysis: Eye movements

Eye movement calibrations were evaluated for accuracy at the beginning and end of the task. A median of 10 points were tested both before and after the task: owing to problems with the calibration/validation target sometimes being outside the scene camera field of view, the number of points available ranged from 3 to 15. For each validation point, an annotator paused the video when the eye fixated and marked the horizontal and vertical coordinates of both the eye gaze cursor and the validation point. The median accuracy (Euclidean distance between the target and point of gaze cursor) was calculated across the points per individual. The pretrial average median error across included subjects (see inclusion discussion elsewhere in this article) was *M* = 1.8° (*SD* = 1.1°; range, 0.3°–4.3°). The post-trial average median error was *M* = 2.9° (*SD* = 1.9°; range, 0.6°–6.3°). Three participants had post-trial errors of greater than 5°, but our inclusion threshold used the pre- and posterror average, which was less than 5°.

LAFs were annotated using CinePlay software by marking the begin and end time of the fixation(s), the current ongoing task and the target of the fixation(s). Note, a participant may make several consecutive fixations on the same object, interspersed with small eye movements. Such consecutive fixations were grouped together and treated as a single LAF. In other words, an LAF is recorded as any set of one or more consecutive fixations on an object. In the event of multiple fixations, the begin time marks the beginning of the first fixation and the end time marks the end of the last fixation. When the participant returned later to interact with that object, the time when the participant touched the object was recorded and the time when the guiding fixation began. Note, if an LAF is made and the object is subsequently touched multiple times, the latency is computed from the end of the fixation to only the most immediate future timepoint when the object is touched; that is, one LAF can be associated with one future touch event.

The annotation for the ongoing task followed the labelling convention for subtasks. Possible LAF targets included the vent cover, tent top, tent corner, tent bag, tent, support tab, support bag, support, stake bag, stake, instruction, and guyline. Instructions are special in that reading can take place with or without picking up the instructions, but participants may also look ahead to them to get position or orientation information. To exclude moments of obvious reading, we only coded single fixation instances to the instructions; series of fixations were excluded owing the confound with reading. Fixations on the tent categories were judged liberally, that is, fixations generally near the tent corner (within approximately 3°) were labelled “corner,” and fixations near the tent top were labelled “tent top.” Any other fixations on the tent were simply labelled “tent.” During initial phases of assembly, the guylines are usually on top of the tent in a rather messy fashion, sometimes leading to ambiguity on whether a fixation was on the guyline or tent itself. Similarly, we used a liberal criterion where fixations with approximately 3° were marked as on the guyline as well as taking into account recent history, that is, a series of fixations on the guyline with one slightly off the guyline would be marked together as one look ahead segment. Similarly, a liberal classification approach was taken to when a LAF interval was complete; for instance, a LAF to the tent corner was ended by any touch made near the corner, regardless of whether the participant then inserted the support, staked the corner, or simply moved the tent. We annotated LAF data as objectively as possible by relating fixated objects to the tasks where they were manipulated manually. However, we cannot exclude the possibility that some LAFs were associated with the wrong task (e.g., some LAFs could have been gathering information for immediate use in navigation or another behavior we had not considered).

### Analysis: Participant inclusion

Participant inclusion varied across analyses owing to varying data quality. All 23 participants answered the experience questionnaire and had their videos annotated for subtasks (using the SMI scene video with point-of-gaze overlay supported with GoPro video as needed; discussed elsewhere in this article). However, for eye tracking analysis, three participants’ eye tracking data were too poor to be included in any relevant analyses (>5° averaged pre- and post-trial error). Additionally, with two participants the eye tracker stopped during recording, leaving only a partial record. This left 18 participants with good quality data to be included in the eye movement analyses.

## Results

The results are organized as follows. First, we present a summary of our participants’ prior experience with assembling camping tents. We then describe general strategies and behaviors used by our participants with further analysis spent on subtasks. Last, we describe eye movement behavior focused on LAFs.

### Behavioral performance

Participants generally had a low to medium amount of prior experience setting up the tent. Nine of the 23 participants owned a tent, with a median frequency of 0.5 camping trips per year. Fourteen participants described their camping experience as low, six medium, and three high. Last, the estimated number of tents setup in a lifetime was a positively skewed distribution varying from 0 to 30, with a median of 4.5.

We made a composite experience score by normalizing by maximum response for each question and then averaging across questions (equal weighting across), resulting in a single value between 0 to 1 that quantifies experience. This results in a right-skewed distribution (*M =* 0.27; *SD =* 0.27; median = 0.16; range = 0–0.81).

Participants took between 5.3 and 24.1 minutes to complete the task (*M =* 12.3 minutes; median = 11.4; *SD =* 4.6). All participants were able to erect the tent, albeit sometimes with slight problems, such as forgetting the vent cover, not tying the support beams to the top of the tent, or staking the guylines incorrectly. Note that self-rated experience did not significantly predict duration, *R^2^* = 0.11, *F*(1,21) = 2.59, *p =* 0.12. Additionally, the experience score did not significantly predict total time spent reading instructions (see 4.1.2), *R^2^* = 0.06, *F*(1,21) = 1.3, *p* = 0.27.

### Subtask sequences

**Figure 2.**
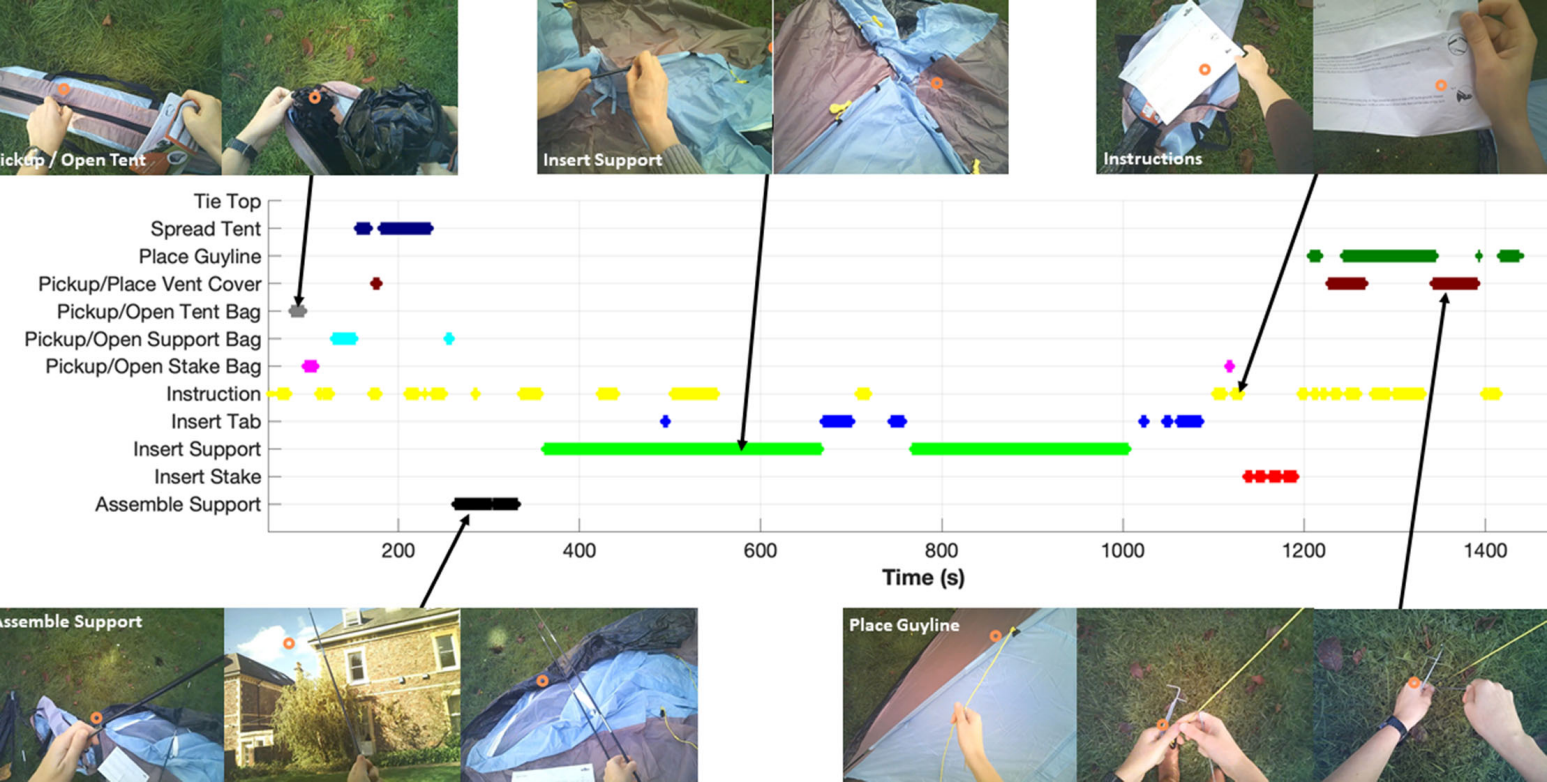
Timeline of events for a single participant. Screen shots display examples of a subset of the subtask categories. The orange circle represents the eye tracker point of gaze estimate.

**Figure 3.**
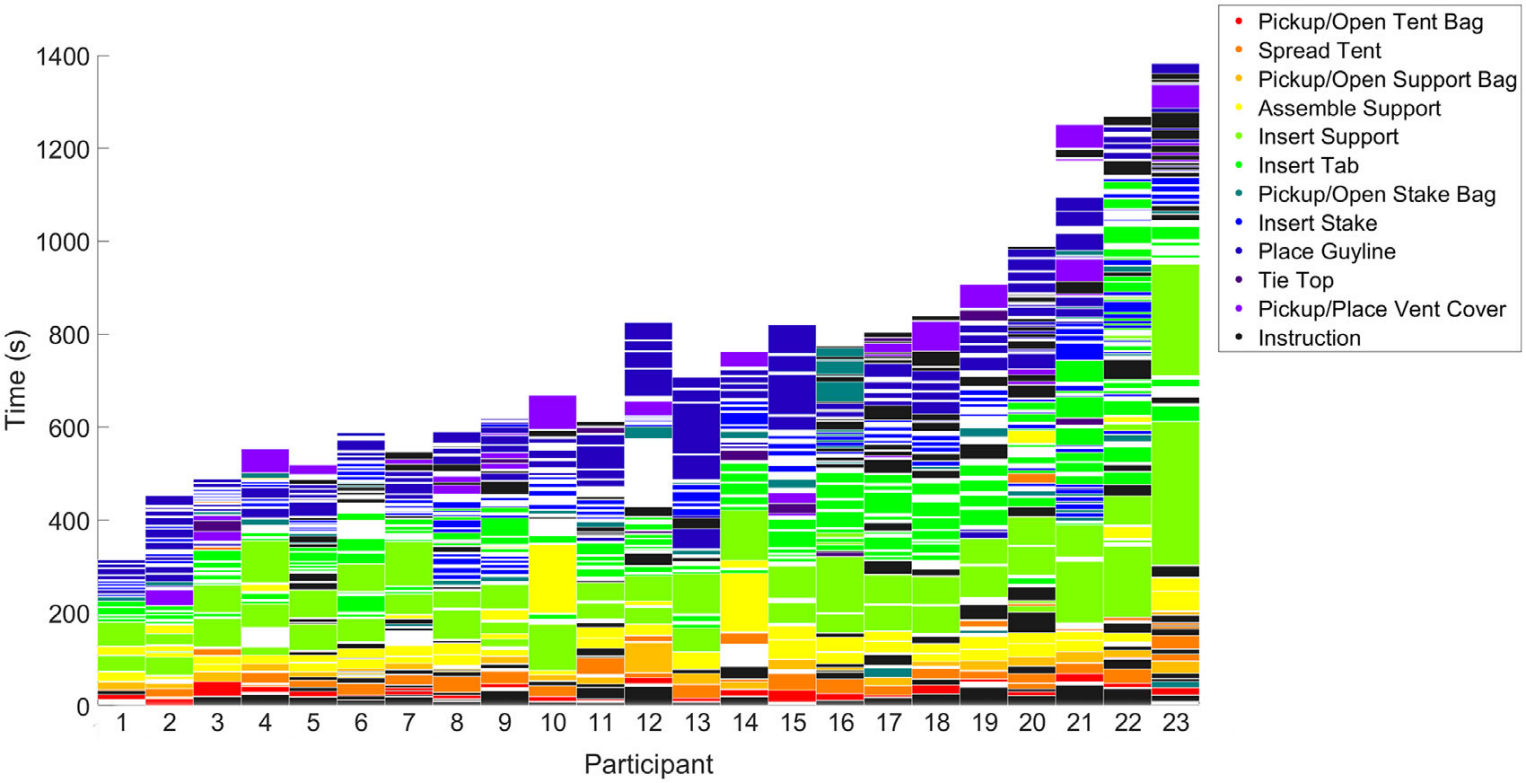
Subtask sequences for all participants. Color bands indicate period when a participant was engaged in a particular subtask. Participants are organized from left to right according to trial duration. Note most whitespace gaps indicate moments of approach transitioning between tasks, extended gaps indicate times of experimenter intervention to answer questions or adjust cameras or equipment. Note that participants 1, 3, 13, 18, and 20 were not included in the eye movement analyses owing to poor data.

Although our primary aim is to understand visual behavior, given the complexity of the visuomotor sequences required for this task it is useful to first consider participants’ actions while assembling the tent, after which we detail eye movement behavior. ^1^Figure 2 illustrates the time series of subtasks for a single participant with example screenshots from selected subtasks. We take this type of time series representation and plot the sequence of subtasks sorted by trial duration for all participants in Figure 3. Using rainbow coloring, this graphic demonstrates how similar the subtask sequences are across participants, but also reveals common differences in sequencing (e.g., some participants assemble both supports in a row and then insert them, whereas others assemble one and insert and then assemble the second and insert). Similarly, common omissions like not tying the top or placing vent cover are visible.

Subtask durations have a wide range (see Appendix Table A1). Reading instructions, insert support, insert tab, and place guyline exemplify high variance subtasks (*SD* *≥* 67 seconds), whereas picking up/opening the tent bag, spreading the tent, and picking up/opening the support bag exemplify low variance (*SD* ≤ 14 seconds). To examine if any particular subtask acted as a performance bottleneck, a linear regression analysis was conducted per subtask using each participants’ subtask durations as the predictor of overall trial durations, with the particular subtask's contribution subtracted from overall duration. Bonferroni corrected tests (α = 0.004) show that the spread tent task, *F*(22,1) = 16, *p =* 0.001, *R^2^* = 0.43, *β =* 12.4 seconds, and the read instruction task, *F*(22,1) = 11.7, *p =* 0.003, *R^2^* = 0.36, *β =* 1.5 seconds, both predicted increased trial duration. The insert tab task was marginally significant in predicting trial duration, *F*(22,1) = 10.2, *p =* 0.004, *R^2^* = 0.33, β = 2 seconds. Therefore, it seems that it is not simply the case that slower participants are slower in all components of the task.

**Figure 4.**
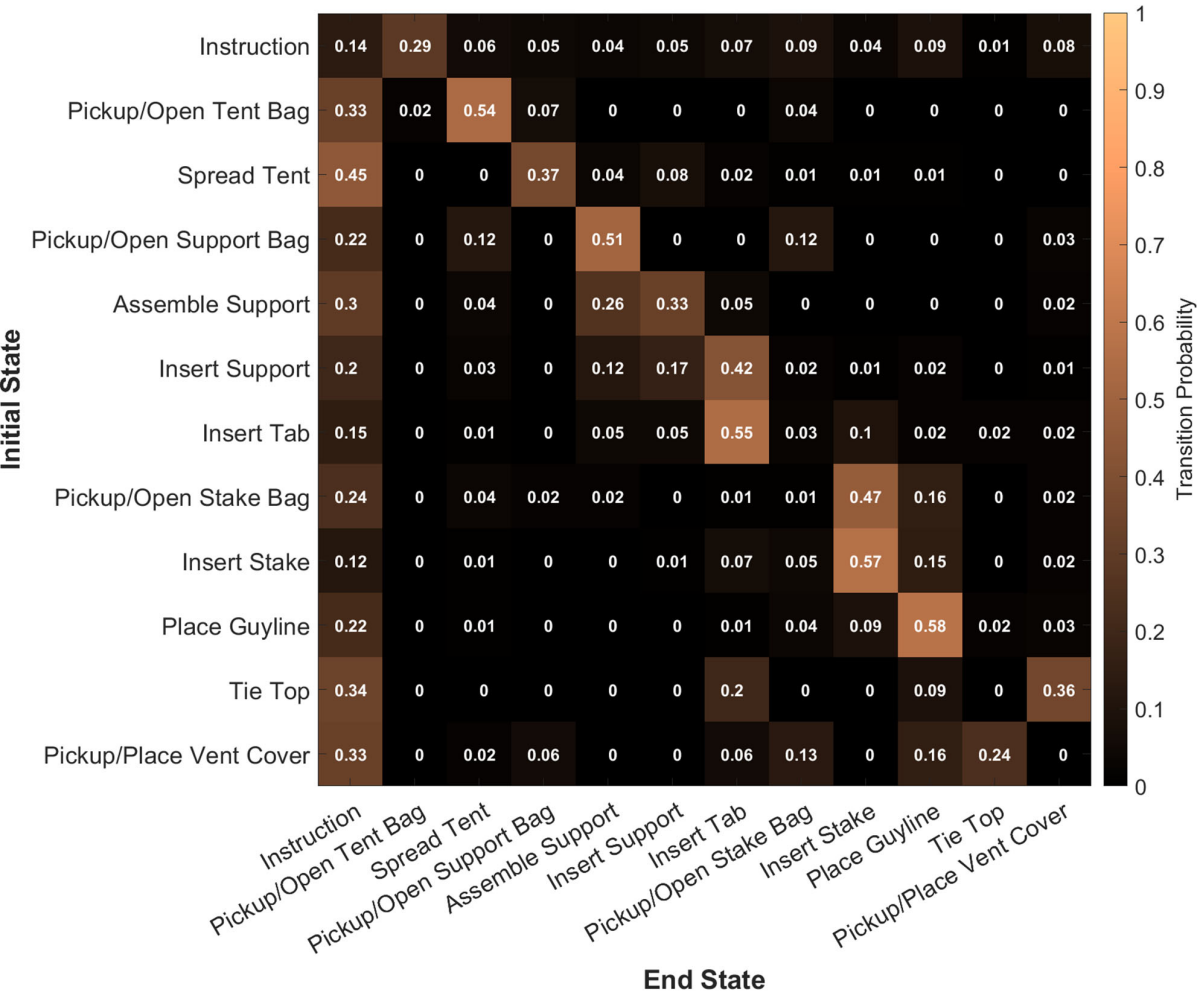
Mean transition probabilities between subtasks. For each subtask, the transition probability of moving from the subtask on the vertical column to the ending subtask along the horizontal is represented by the color. Transition matrices were calculated for all 23 participants and averaged.

Although all participants were eventually successful in setting up the tent, the ordering of their behavior varied. To capture the sequence of behaviors, subtask event labels can be treated as states in a Markov transition matrix, as shown in Figure 4. A transition matrix was calculated per participant by counting each time they shifted from one task state to another and then averaged across participants. These probability transitions are useful as a descriptor of behavior, because they delineate likely and unlikely behavior sequences. As one might expect, the transitions largely follow the order of the instructions in Table 1, but there are several deviations.

The bulk of the high probabilities are along the diagonal shifted over by 1, as the task labels are ordered in an idealized chronological sequence and show the strong tendency for this sequence. Note, there is no single correct way to build the tent and there are many lower probability transitions showing the diversity of assembly sequences. The exceptions to the shifted diagonal are insert tab, insert stake, and place guyline, all tasks that typically are done in sequence with four repetitions. Across all subtasks, the only end state with all nonzero probabilities is reading instructions, indicating participants may consult the instructions at any time during the task.

The transition matrix allows the calculation of the probability of a participant's particular subtask sequence against a reference transition matrix. Assembly sequences closer to the reference will have a higher probability compared with unusual sequences, possibly identifying participants having trouble. We examined the relationship between log probability of a participant's path compared with an idealized perfect path (a Markov matrix constructed around the sequence in Table 1 with small values of 1^–^^6^ inserted in place of zero probability transitions). A linear regression was conducted using log probability as a predictor of trial duration, and showed that a lower log probability predicts increasing duration, that is, unlikely paths also take longer, *F*(22,1) = 9.8, *p =* 0.005, *R^2^ =* 0.32, *β = −*1.2 seconds. However, a linear regression using log probability as a predictor of composite experience level did not have a significant relationship, *F*(22,1) = 0.01, *p =* 0.91, *R^2^ =* 0.001*,* nor did any individual component of the experience questionnaire. This finding suggests that our self-declared novices were relatively competent and that our “experienced” participants may have overestimated, or their expertise was disrupted by using an unfamiliar tent. It could also be that tent assembly is so infrequent that even experienced participants’ past knowledge does not generalize to a novel tent.

### Eye movements and LAFs

During tent assembly, visual behavior is largely occupied with guidance as participants monitor the state of various parts as they move them into place. Participants also read the instructions and search for items (usually briefly) as needed. Qualitatively, as observed during annotation, few fixations were on task irrelevant objects and search toward task-relevant objects was brief, usually done in 1 to 3 saccades.

LAFs were marked for the 18 participants with valid eye tracking data, detailing the object of the LAF, the ongoing task and the time when the participant returned to manipulate the object. Overall, 739 LAFs were annotated. On average, participants made *M =* 41 LAFs (*SD =* 29.9; range = 5–105 LAFs). The LAF duration distribution is right skewed (*M =* 583 ms, *SD =* 206 ms; Appendix Figure A4). Recall that our definition of a LAF can consist of consecutive fixations, so the LAF duration should be thought of as a summed dwell time on an object that is later interacted with. The number of LAFs increased with longer assembly durations, *R^2^ =* 0.58, *F*(17,1) = 8.5, *p =* 0.01. However, the LAF rate is stable across participants, LAF rate per minute across participants (*M =* 2.7; *SD =* 1.4), has no significant relationship to trial duration, *R^2^ =* 0.02, F(17,1) = 0.28, *p* = 0.6. The number of LAFs also does not have a significant predictive relationship with self-rated experience, *R^2^ =* 0.04, *F*(17,1) *=* 0.8, *p* = 0.4.

### LAF latency interval

**Figure 5.**
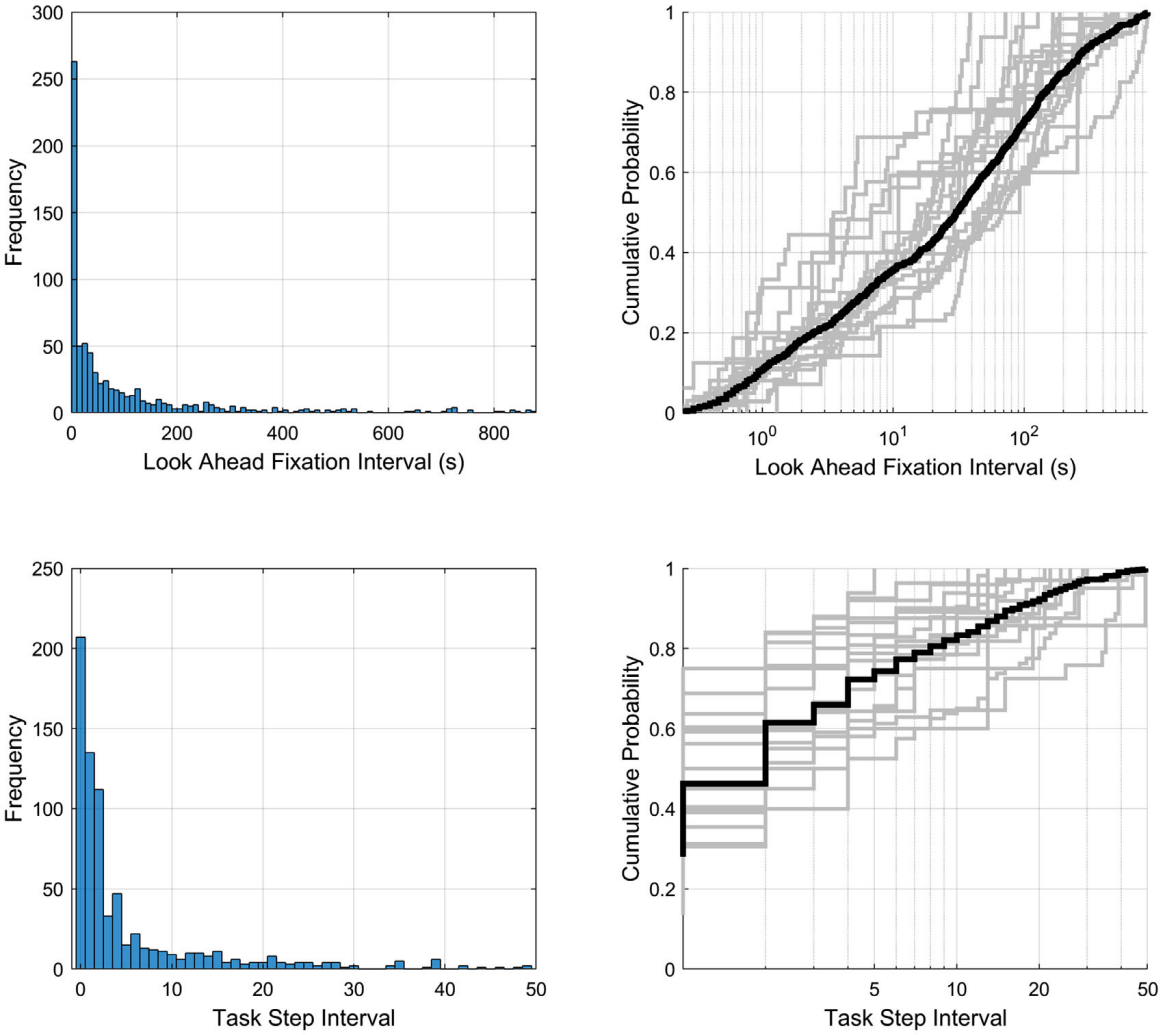
Frequency distribution and cumulative probability of LAF intervals. (Top left) Frequency of LAF intervals in absolute time (seconds), bin size of 10 seconds. (Top right) Cumulative probability of LAF intervals with log *x*-axis. (Bottom left) Probability of LAF intervals in subtask steps. (Bottom right), bin size 1 step. Cumulative probability of LAF intervals in subtask steps.

The LAF interval distribution, that is, the time between the end of the LAF to the beginning of the guiding look associated with manipulation, is shown in Figure 5 (top) along with the cumulative probability across intervals. The peak LAF latencies are less than 5 seconds, but the tail of the distribution is long. We find that about 35% LAFs occur within 10 seconds (the typical range of prior findings), about 40% occur between 10 and 100 seconds, and the remaining 25% at more than 100 seconds (maximum of 880 seconds). When a participant begins the assembly and removes all items from the tent bag, this action creates many opportunities for early lookaheads, as assembly progresses opportunities arise but decrease as assembly nears completion. Importantly, as shown by the cumulative distributions for individual participants (in grey), we observed this wide range of variation in LAF latencies for every single participant. That is, even the participant with the shortest LAF latencies still had several more than 10 seconds and the same holds true for the one participant with only five LAFs. Appendix Figure A1 plots the aggregate distributions of LAF latencies, separately for each subtask during which the LAFs were made, and shows that latencies vary dependent on the ongoing task and almost all have long latencies or more than 10 seconds and most of more than 100 seconds. This finding suggests that some ongoing tasks may favor shorter latency LAFs than others, but long latencies are not exclusive to a particular subtask, such as reading instructions, where participants might briefly pause reading to make LAFs to relevant objects.

The LAF latencies as discussed are much greater than previously reported, and one might argue that many long LAF latencies are incidental. We addressed this issue in two ways. First, it could simply be that objects with longer time to use (the amount of time from the trial start to the time that a LAF target was used) have longer latencies because there are more opportunities to fixate the object incidentally before its use. Given that we have a much longer task, it would therefore not be surprising that we observed longer LAF latencies. To examine this possibility, we ran a linear regression using time to use to predict LAF latencies. Indeed, we found a significant increase in LAF latency predicted by increasing time to use, *R^2^ =* 0.13, *F(738,1) =* 107, *p =* 1.6^–^^23^, *β =* 0.2 seconds. However, the variance explained is relatively small, suggesting that although some long LAF latencies may be due to some objects having a long time to use, it is not dominant.

Second, we reasoned that LAFs made to objects relevant to the current ongoing task (see LAF task and target dependence) are less likely to be incidental compared with LAFs directed to upcoming tasks. This assumption may be overly conservative, because it excludes the many LAFs related to upcoming tasks that are necessarily further ahead in the future (Figure 5 and LAF task and target dependence), but may be a more reliable estimate. We compared on- versus off-task LAF latency distributions, and found that while both contain longer latencies than prior studies, on-task latencies are significantly shorter *(M =* 37.6 seconds*;* median *=* 6.5 seconds*, SD =* 83.7 seconds) than off-task *(M =* 136.1 seconds; median *=* 54.2 seconds*;* *SD =* 197.6 seconds), *t(445) =* 6.7*,* *p =* 7.4^–^^11^). Importantly, the mean latency of 37.6 seconds for on-task LAFs is much greater than the mean of 2.5 seconds reported by Mennie et al. (2007), likely owing to the vastly different tasks performed.

We also analyzed this distribution considering task steps instead of absolute time as this may be more directly related to the planning process. Figure 5 (bottom) shows that approximately 75% of LAFs are made to objects within five task steps, 90% within 15 steps, with the remainder to objects up to 50 tasks steps ahead. Like absolute time, LAF latency as a function of subtask steps is right skewed and similarly distributed across participants. Appendix Figure A2 plots the aggregate subtask step distribution per LAF target object, and, given the tight relation between objects and subtasks, shows again that latencies are task dependent.

In summary, we observe much longer and more variable LAF latencies compared with previous studies. Although some LAFs are no doubt incidental, it seems likely that some long-latency LAFs are purposeful, given the limited predictive power of time to use and the long latencies observed even when we restrict our analysis to on-task LAFs.

### LAF task and target dependence

**Figure 6.**
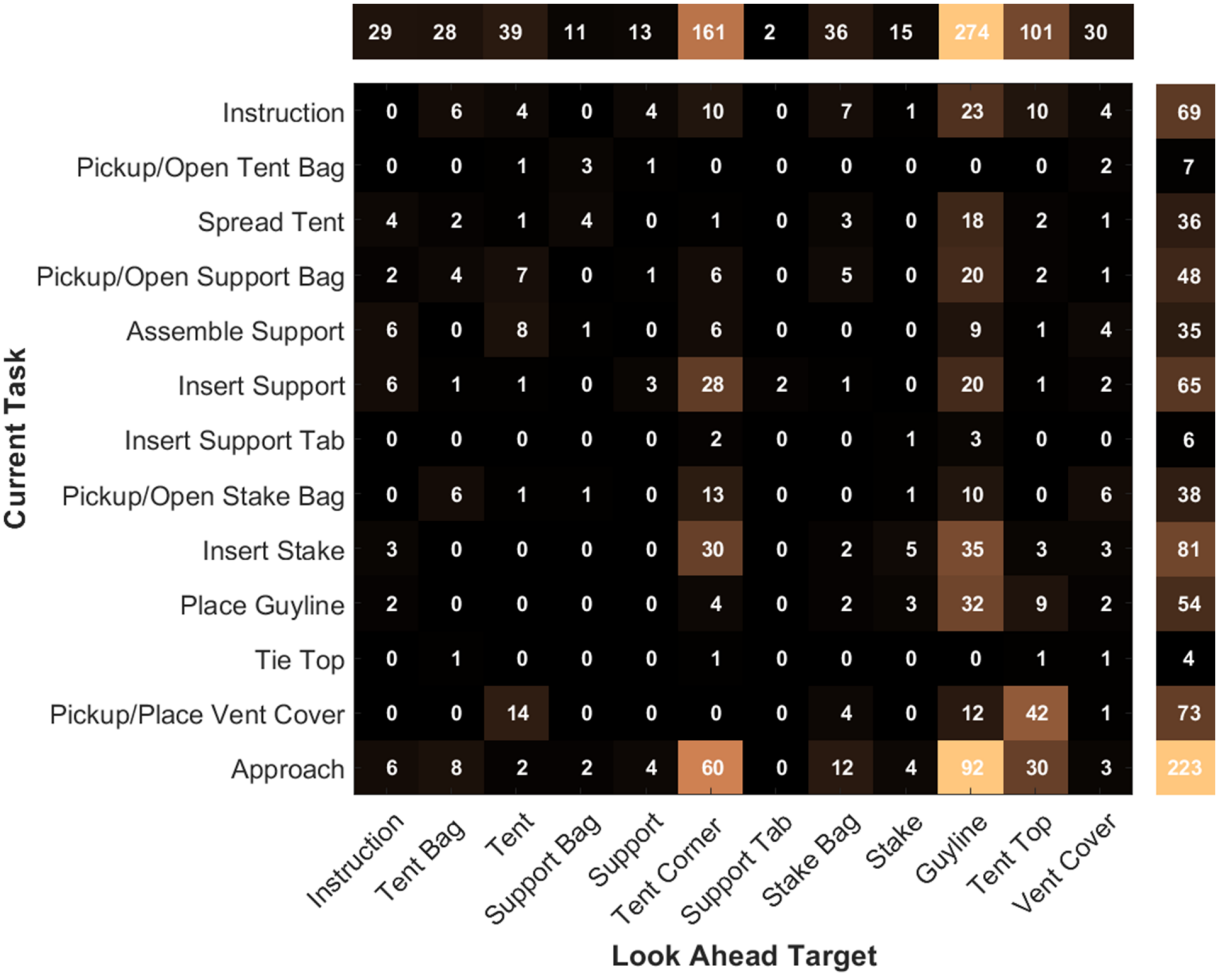
Frequency of LAF targets and ongoing tasks. Rows depict subtasks and each column entry indicates the frequency of LAFs to a target object during that task. The solitary row above indicates the sum of LAFs per target. The solitary row to the right indicates the sum of LAFs per subtask.

Prior research has demonstrated LAFs are sensitive to task demands, and Figure 6 shows LAFs frequencies aggregated across participants per subtask and per object. The summed raw frequencies in the margins are per subtask (right) and per target (top).

If we only consider ongoing subtasks (right margin), some were more likely to have a LAF occur, with the most common being during approach (30%), insert stake (11%), and pickup/place vent cover (10%). Averaged across participants, we find the number of LAFs made during each subtask ranged from near zero (pickup/open tent bag, insert support tab, tie top) to between two and five LAFs (instruction, spread tent, assemble support, insert support, pickup/open stake bag, and insert stake), with most occurring during approach (*M =* 12; Appendix Table A2).

If we only consider LAF targets (top margin), we find LAFs were biased toward some objects, with LAFs most commonly being made to the guyline (37%), tent corner (22%), and tent top (14%). Averaged across participants, the number of LAFs varied per target ranging from 0 to 15 LAFs (Appendix Table A3). Some targets get less than one (support bag, support, support tab, and stake) and others one to two LAFs (instruction, tent bag, tent, stake bag, vent cover), with a few getting frequent LAFs: tent corners (*M =* 8.9), guylines (*M =* 15.2), and tent top (M = 5.6).

The individual cells of Figure 6 depict the frequency of LAFs to each target for each ongoing task. LAFs may be directed to targets relevant to the ongoing task or an upcoming task. For example, when inserting a stake to secure the tent corner, two common LAF targets are the tent corner (relevant to the current subtask) and the guyline (relevant to an upcoming subtask). We examined if LAFs tended to be made to objects related to the current task or to a future task. Appendix Table A4 explains how on- versus off-task LAFs were determined. Note, approach and instruction subtasks are excluded from this calculation because all LAFs in these subtasks are by default concerned with upcoming tasks.

Averaged across participants, a mean of 47% (*SD =* 17%) of LAFs are made to the objects within the current task. Furthermore, certain tasks are more likely to generate within task LAFs; for instance, more than 60% of LAFs made during the pickup/open tent bag, pickup/place vent cover, place guyline, and spread tent tasks are made to objects relevant for that task (Appendix Figure A3).

### Repeat LAFs

Do participants make just one LAF to a target before interacting with it, or do they sometimes make repeat LAFs revisiting the same target multiple times? Repeat LAFs could suggest information obtained from a LAF is volatile owing to decay or dynamics in the world. Of the 739 LAFs we identified, 183 were single, isolated fixations by an observer to those 183 targets. The remaining 556 LAFS consisted of two or more LAFs each to 161 other targets. Averaged across participants, 45% of LAF targets were revisited (*SD =* 16%). The mean time difference between repeat LAFs was a mean of 44.5 seconds (*SD =* 77.5 seconds); the median was 14.1 seconds.

The most common repeat LAF sequences were two (53%), three (23%), and four (10%). Multi-LAF sequences were target dependent, occurring primarily for the guyline (25%), tent corner (27%), and tent top (13%); all other targets were at or below 6%.

## Discussion

We present behavioral data from a group of participants assembling a camping tent. Participants had a wide range of prior experience, but all were able to successfully complete it. At least in this sample, self-reported experience did not seem to predict behavior; rather, the time taken to complete the task was predicted by the time spent on particular subtasks. Using an annotation of actions, we show that, although participants exhibit variety in their behavioral sequence, there are core common elements shaped by task demands. The likelihood of a participant's trajectory through the state space, as captured by a Markov transition matrix (Figure 4), is predictive of the duration of assembly, but not the composite experience score.

Eye movement behavior largely consisted of sequences of brief search for an item, followed by guidance to manipulate the item. An extended search of longer than 1 second was infrequent, and participants largely made fixations only on task-relevant objects.

We document four novel aspects of LAF behaviors: (1) diversity across objects and tasks, (2) variance in latencies much greater than previously noted, (3) LAFs can be framed in terms of task steps and 53% are to targets off the current task, and (4) LAFs often have one or more revisiting fixations to the target before manipulation.

LAFs can have long latencies, with 80% being made within 100 seconds and the remainder having even longer latencies. Reframed in terms of subtask steps, one-third of LAFs are relevant to the current task and most of the remainder are to subtasks up to 15 steps away. Setting up a tent has several unique features that may explain these results: the spatial layout is highly varied, starting with all items in a single bag until ultimately spread out over about a 2 × 2 m area. Setting up a tent takes at least 37 idealized steps, requires frequent walking around the environment and the moving of multiple items. This process creates a situation where participants may frequently be uncertain about the appearance and location of multiple objects as the task evolves, and LAFs could reflect one way to decrease this uncertainty in anticipation of upcoming actions.

The frequency of LAFs varies across participants and is predicted by trial duration. LAFs are also more common in certain subtasks (independent of duration) and certain items are far more likely to be the target of a LAF. Tasks likely to generate a LAF include approach, where the hands are unoccupied and the participant is moving around the tent giving opportunity to view many objects, and insert stake, where participants’ hands are busy pushing down. The most common items to be target by a LAF are the tent corner, which is relevant during insert stake, insert support, and insert support tab, and the guylines as described elsewhere in this article. Finally, LAFs were often made in a series, with 47% of LAFs having two or more fixations before object interaction, suggesting memory volatility either owing to internal decay or world dynamics.

Latencies similarly vary according to the LAF target object and the ongoing task. For example, during instruction reading, participants sometimes pause reading and look around, leading to a large variety of latencies. However, during pickup/place vent cover, most LAFs are directed to the corners of the tent top that will secure the cover and yield a distribution biased toward short latencies. However, the ultimate purpose of long latency LAFs are difficult to identify from an observational study because the LAF and the action it may serve are so distant in time.

Why do we measure LAFs with longer latencies than prior studies? First, LAFs have not been frequently addressed leading to a sparse research history. Additionally, owing to the flexible nature of vision, LAF research in walking, driving, and playing music has focused on short LAFs that seem to be dedicated to gathering information in the visual buffer for actions within the next few seconds. The longest LAF noted before this study (68 seconds) was an anecdotal mention by Land et al. (1999) while making tea. Other studies like Pelz and Canosa (2001) and Mennie et al. (2007) had tasks intentionally structured to last only 1 to 2 minutes; further, the amount of information available for future interaction was limited, whereas in our task there are at least 37 steps with 12 task-relevant objects. Making tea is the most similar in terms of task complexity and one might speculate there were other long latency LAFs that were undocumented. We also analyzed LAFs targeting on-task versus off-task objects under the assumption that on-task LAFs may contain fewer incidental fixations. On-task LAF latencies were, naturally, much shorter on average than off-task. However, they were still much longer than prior studies, suggesting that the range of latencies is larger than previously known, even when we only consider more reliable on-task LAFs.

The role that LAFs play in natural tasks is unclear, and it is difficult to know which are purposeful and which are incidental. Prior studies have found mixed evidence for the quality of information taken from preview fixations and that incidental fixations do not help performance (Võ & Wolfe, 2012; Kit et al., 2014). However, there is some evidence that LAFs can improve saccadic accuracy (Land et al., 1999; Mennie et al., 2007).

We can only speculate on the usefulness of LAFs made by our participants, but watching their eye tracking videos provides many clues. For example, when attaching the vent cover, which has four individual hooks that need to be attached, participants routinely look to the next attachment location on the tent top while fixing the hook in place at the prior one, a very clear on-task LAF. Similarly, when inserting the stake in the tent corner, a participant may make an on-task LAF to the next corner they will stake. This finding may suggest that LAFs are biased toward gathering information for tasks that require more precision or manual dexterity. However, not all examples are easy to interpret. For example, while walking around the tent during approach, participants often look at the guylines or tent corners. These LAFs may be relevant for upcoming tasks because the participants must interact with guylines and corners at least four times, but they could have other explanations. The guylines are long bright yellow ropes and may be a salient target in contrast with an otherwise uniformly colored tent. Tent corner LAFs may serve navigation around the tent or help in evaluating if the supports or stakes are secured.

Despite being outdoors in a garden, nearly all of participants’ fixations were on task-relevant objects; if some LAFs are incidental, they may be due to attentional biases owing to future relevance. Although they did not model visual attention, using Cooper and Shallice's (2000) framework, one might imagine incidental LAFs arising owing to schema activations sufficient to shift visual attention but insufficient to initiate action. However, given the task dependence and object dependence of LAFs and the tendency to be on task or a few steps ahead, it seems unlikely that most LAFs are incidental noise. Future experimental studies of this topic should either account for, or control, the visibility of objects and the angle subtended, because larger objects may be more likely to be fixated simply by virtue of their size.

Land and Furneaux's (1997) study of look ahead behavior highlighted the idea of a visuomotor buffer capturing information about 1 second ahead as part of a visuomotor control loop for tasks like driving and sight reading music. Mennie et al. (2007) documented a model assembly task with peak LAF latencies of 2.5 seconds. Land et al. (1999) anecdotally described longer latencies in tea making like our observations. Given the variance observed in LAF latencies during tent assembly, it seems unlikely that long latency LAFs (>100 seconds) have the same purpose as those with short latencies (<10 seconds). Considered in terms of task steps, LAF latencies suggest a mix of short-term and longer term planning, with about one-third of LAFs made to objects related to the current task, and the remainder to one or more steps beyond the current subtask.

The frequency of repeat LAF sequences, that is, more than one LAF to the same object before interacting with it, occurred 47% of the time. This finding suggests that, if most LAFs are purposeful, the quality of information extracted is coarse or not well-maintained in memory and may reflect the flexible trade-off between memory and saccades seen in other tasks (Ballard, Hayhoe, & Pelz, 1995).

We hypothesize that LAFs are generated by three processes: (1) action driven (short latency <10 seconds): Coinciding with most of the previous study of LAFs, these LAFs extract visuospatial information and mainly influence motor behavior (hand motions, eye movements, and moving the body in space) and would contain fixations related to Land's visuomotor buffer. This category might be further divided to distinguish control loop LAFs (<1 second). (2) Cognition driven (long latency >10 seconds): Purposeful strategic LAFs that may be part of planning the next steps or checking a mental inventory of task-relevant objects. Owing to long latencies, visuospatial information acquired is poor but sufficient for planning, for example, “the parts needed for the task step a minute from now are over to my left.” (3) Task relevance and salience: “Incidental” LAFs of any latency made to objects owing to task relevance, or a task-relevant object having salient image features, but not otherwise purposeful.

These hypotheses need to be vetted experimentally because LAFs presumably are a probabilistic mix of the above LAF types, other processes, or could be further subdivided (e.g., short latency LAFs for motor information vs. short latency LAFs for problem solving). Gaze-contingent virtual reality setups are well-suited for testing the knowledge gained from LAFs. It should be possible to monitor when LAFs are made to items and alter visual aspects or location and observe any behavioral changes as a function of the LAF latency.

Are LAFs necessary? Are they a default strategy of a visual system used for planning and interacting with a dynamic world while balancing a variety of memory loads? In relation to models of sequential visuomotor behavior, these questions could be approached by modeling the underlying information processing steps in tasks where LAFs are known to occur. One approach would be to consider LAFs in relation to memory and motor coordination using abstract decision models, such as diffusion to bound (Johnson et al., 2014). By considering memory decay and costs for eye and body movements, it should be possible to identify conditions where the information from LAFs improve future behavior. More elaborate approaches using deep reinforcement learning (Vinyals et al., 2019) could simulate a visuomotor agent in an environment where a mapping of visual features to choices for motor selection are learned, and could also vary memory fidelity and motor costs. Such models could identify possible roles for LAFs regarding visuospatial memory, but it is not clear if they can explain more abstract roles for LAFs like problem solving. If the LAFs are a composite of multiple visual computations over varying timescales, simple models might kickstart ideas for experiments that can then inform more complex models. One could adjust systematically the degree of planning, memory load, task and stimulus uncertainty, and on- and off-task salience of in models and experiments to refine our understanding of LAFs.

## Conclusions

In an outdoor tent assembly task, we show that LAFs occur in a wide variety of contexts. LAFs selectively occur more often during certain tasks and to certain objects. Look ahead latencies span short to long timescales and appear to show a mix of on-task and long-term information gathering. Gaze contingent experiments and modelling are needed to delineate how LAFs relate to planning and memory.

## Supplementary Material

Supplement 1

Supplement 2

Supplement 3

Supplement 4

Supplement 5

Supplement 6

Supplement 7

Supplement 8

Supplement 9
