# Supplementary material for: Look-ahead fixations during visuomotor behavior: Evidence from assembling a camping tent
Source: J Vis. 2021 Mar 10;21(3):13. doi: 10.1167/jov.21.3.13 (PMC7961111; doi:10.1167/jov.21.3.13)
Supplement: Supplement 1 [file jovi-21-3-13_s001.pdf]

Appendix

Shared Dataset

We originally presented this dataset as part of ICCV 2019 (Jang et al. 2019). We share: three videos (raw eye tracker scene camera, eye tracker scene camera video with point of gaze cursor, and GoPro video), eye tracker data files, annotation of sub-tasks, errors, and participant frame-by-frame uncertainty rating. Errors were marked in a similar fashion to sub-task labels. Participant self-rating was obtained by having participants watch their GoPro video and rate their level of uncertainty in performing the task using an in-house video viewing program. All data can be found along with further explanation of the dataset at: https://sites.google.com/view/epic-tent

Sub-task Durations

Appendix Table A1 displays the median number of occurrences (for one setup of the tent) and durations of each sub-task across participants. Assuming at least one occurrence of the task took place, the most frequent subtask, 6 occurrences, was inserting the support pole into the tab (there are only 4 tabs, but often participants would miss or have to reconfigure their setup). The least frequent subtasks with a single median occurrence were Pickup/Open support bag and tent bag, spread tent and tie top. Participants spent the most amount of time inserting the support into the tent fabric (u=119s). Participants spent the least amount of time (excluding omitted events), picking up and opening the tent bag (u=13s).

Look Ahead Fixations

When scoring if a LAF was either in-task or out-of-task Table A2 was consulted. We liberally defined what objects were considered on-task and several objects are related to multiple sub-tasks. For example, a LAF on the stake was considered in-task if the individual was currently engaged in: insert stake, place guyline, or pickup/open stake bag.
