# Supplementary material for: Look-ahead fixations during visuomotor behavior: Evidence from assembling a camping tent
Source: J Vis. 2021 Mar 10;21(3):13. doi: 10.1167/jov.21.3.13 (PMC7961111; doi:10.1167/jov.21.3.13)
Supplement: Supplement 2 [file jovi-21-3-13_s002.pdf]

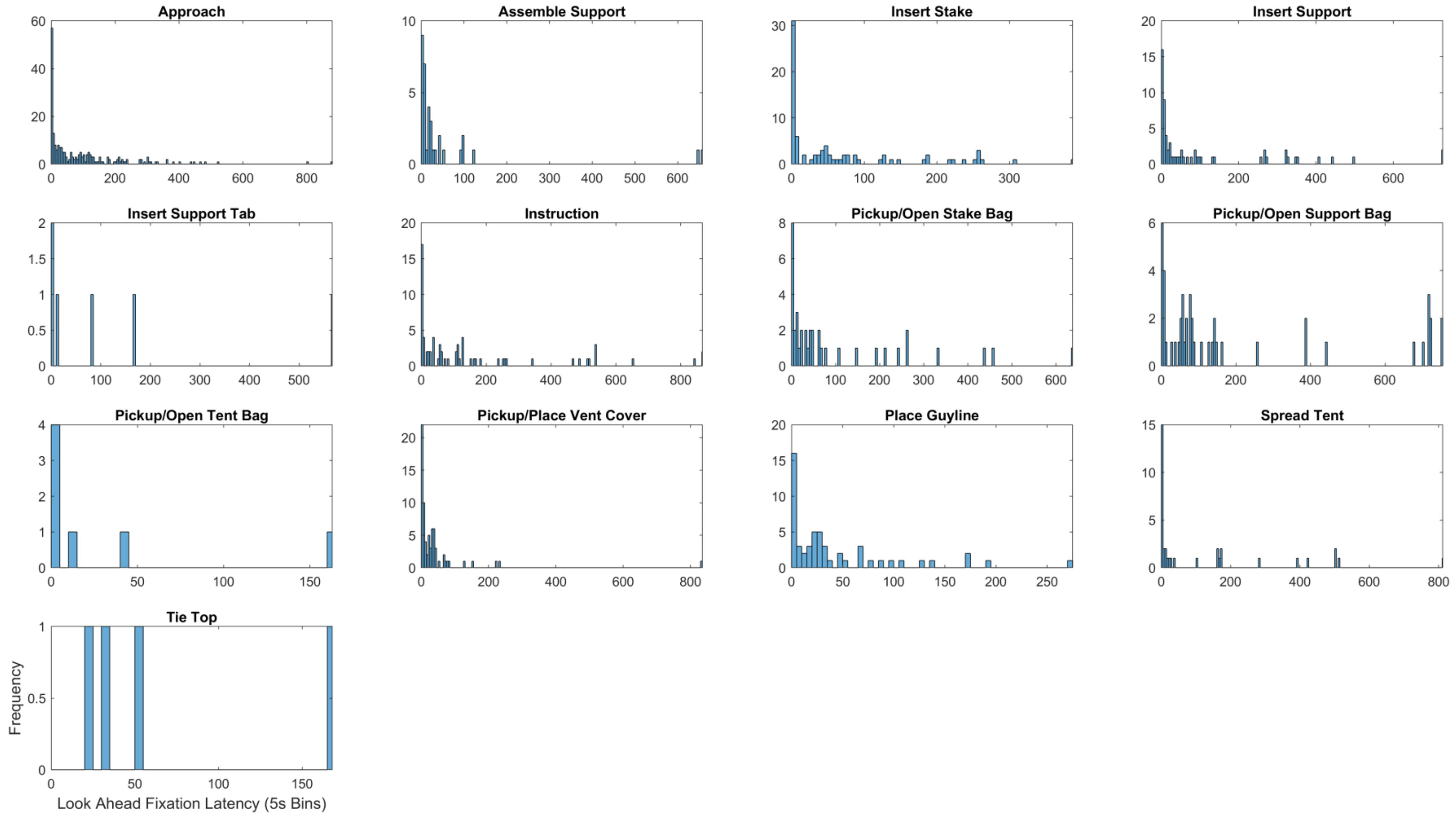

Figure A1. LAF Latency Interval Per Ongoing Task. *The latency between when the LAF was made and when the participant touched the object are depicted sorted by the current ongoing task when the LAF was made. Histogram bins are 5s wide.*
