# Supplementary material for: Look-ahead fixations during visuomotor behavior: Evidence from assembling a camping tent
Source: J Vis. 2021 Mar 10;21(3):13. doi: 10.1167/jov.21.3.13 (PMC7961111; doi:10.1167/jov.21.3.13)
Supplement: Supplement 3 [file jovi-21-3-13_s003.pdf]

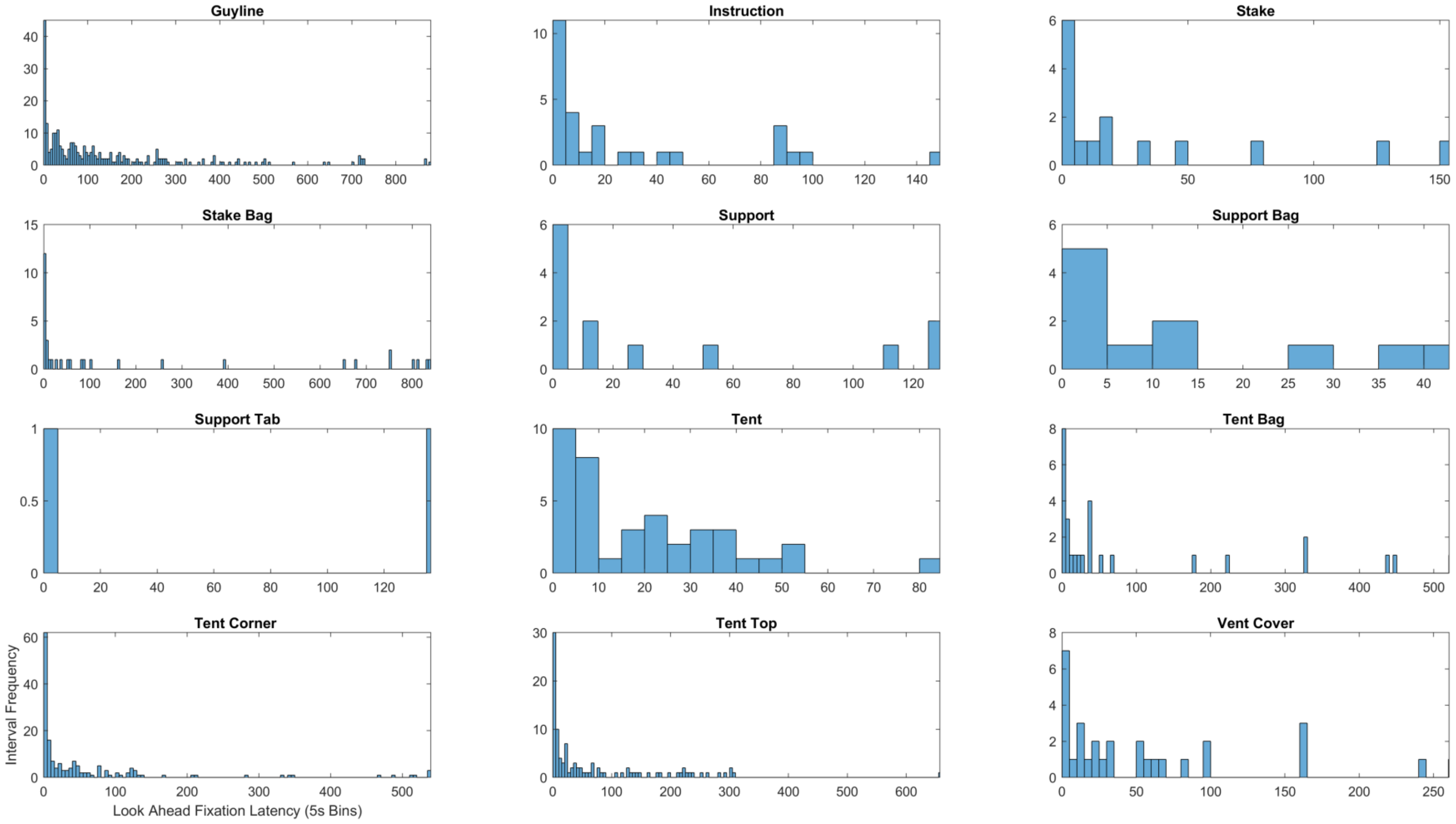

Figure A2. LAF Latency Per Fixation Target. *The latency between when the LAF was made and when the participant touched the object are depicted sorted by the current fixation target the LAF was made to. Histogram bins are 5s wide.*
