# Supplementary material for: Look-ahead fixations during visuomotor behavior: Evidence from assembling a camping tent
Source: J Vis. 2021 Mar 10;21(3):13. doi: 10.1167/jov.21.3.13 (PMC7961111; doi:10.1167/jov.21.3.13)
Supplement: Supplement 4 [file jovi-21-3-13_s004.pdf]

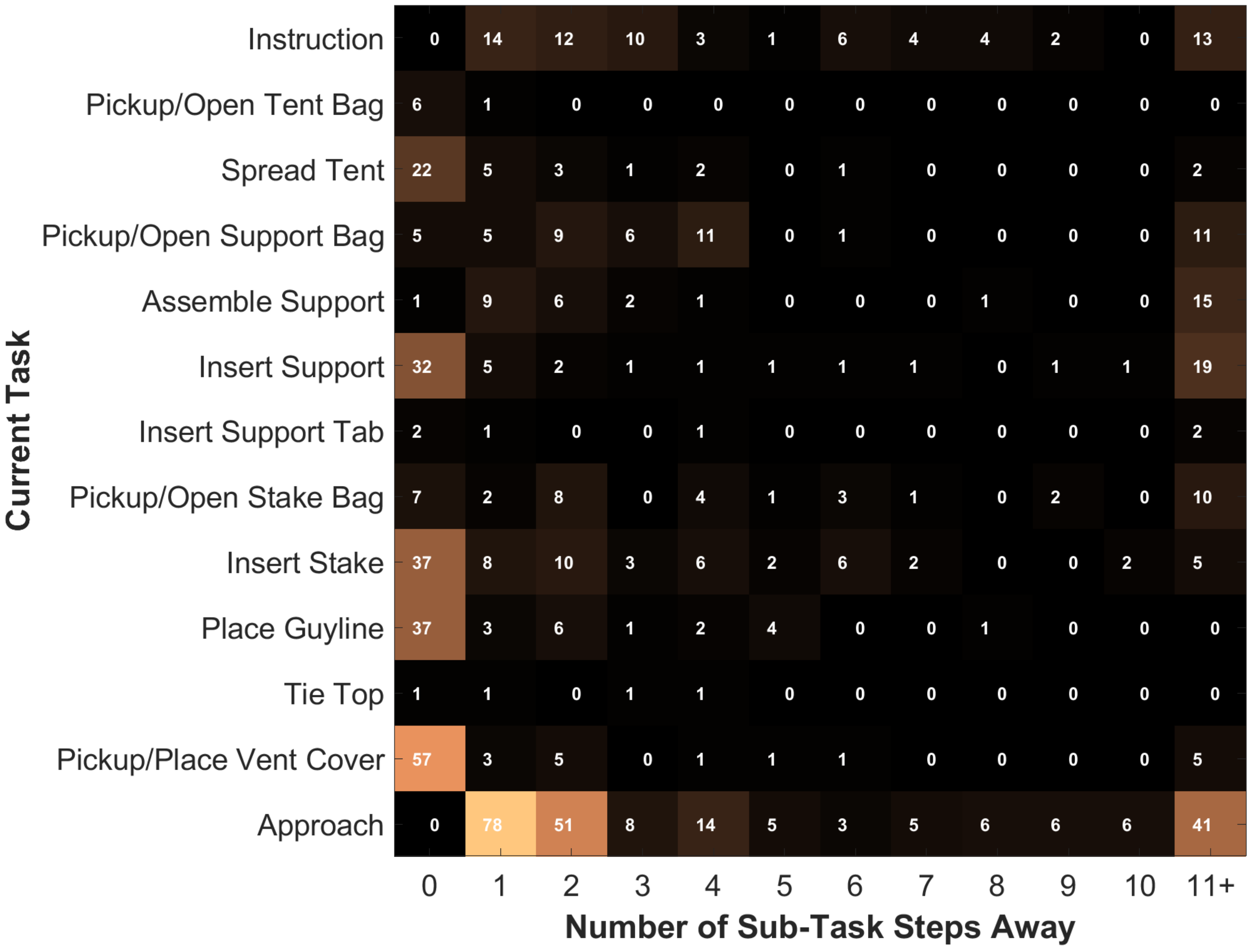

Figure A3. Frequency of LAFs as a function of task-steps. *Rows indicate sub-tasks and each column entry indicates the number of LAFs occurring in that subtask and the number of sub-task steps ahead the LAF was directed. A sub-task step of zero means the LAF was directed within the ongoing sub-task. LAFs are primarily directed to other objects within 10 task steps but can go well beyond (~50 in the extreme)*
