# Supplementary material for: Look-ahead fixations during visuomotor behavior: Evidence from assembling a camping tent
Source: J Vis. 2021 Mar 10;21(3):13. doi: 10.1167/jov.21.3.13 (PMC7961111; doi:10.1167/jov.21.3.13)
Supplement: Supplement 5 [file jovi-21-3-13_s005.pdf]

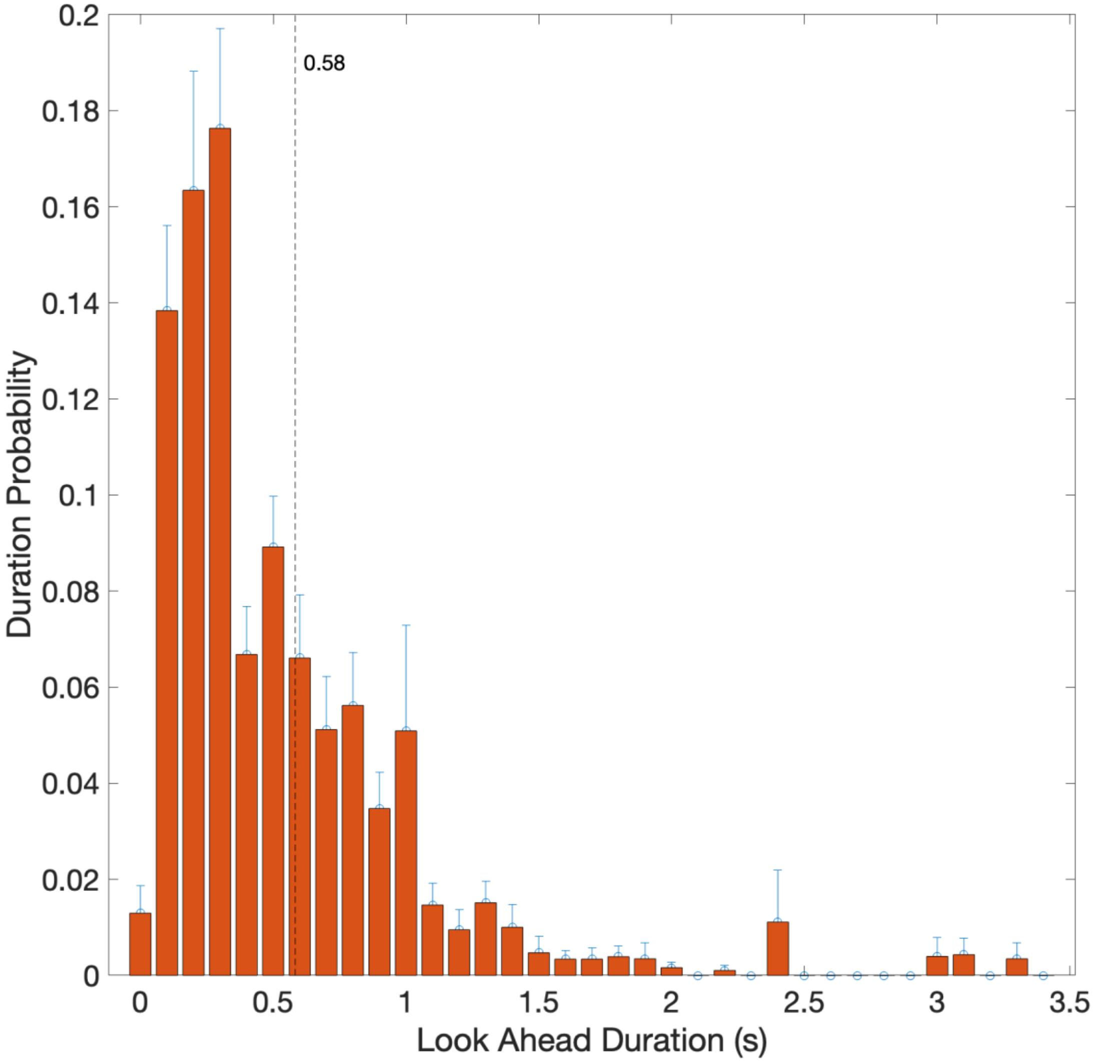

Figure A4. Distribution of LAF durations. Bin size of 0.1s. Error bars in indicate standard error. Note LAFs were annotated as the beginning and end of one or more consecutive fixations on an object, individual fixations were not annotated. Durations are calculated as the time difference between beginning of the first fixation and the end of the last fixation (if there was more than one). The dotted vertical line indicates the mean duration.
